# Low dose IL‐2 suppress osteoclastogenesis in collagen‐induced arthritis via JNK dependent pathway

**DOI:** 10.1002/iid3.364

**Published:** 2020-10-24

**Authors:** Han Sun, Yong Zhao, Kun Wang, Li Zhu, Jian Dong, Jie Zhao, Yimin Wang, Huan Li, Xiaoliang Sun, Yunjie Lu

**Affiliations:** ^1^ The Third Affiliated Hospital of Soochow University Changzhou China; ^2^ Department of Sports Medicine and Adult Reconstructive Surgery, Nanjing Drum Tower Hospital The Affiliated Hospital of Nanjing University Medical School China; ^3^ Department of Hepatobiliary Surgery The people's hospital of Jinsha County China

**Keywords:** c‐Jun N‐terminal kinase, collagen‐induced arthritis, interleukin‐2, NF‐κB, rheumatoid arthritis

## Abstract

**Background:**

Rheumatoid arthritis (RA) is one of the most common chronic immune joint diseases, mainly involving blood vessels and small joints. The complex pathogenesis of RA greatly increases the difficulty of treatment. At present, the common hormone and immunosuppressive therapy are not effective, while low‐dose interleukin‐2 (IL‐2) recently has been found to possess some advantages for immunotherapy. However, its related signal pathway remains to be elucidated.

**Methods:**

We fabricated the model of arthritis in mice, and then low‐dose IL‐2 was injected at a fixed time point to observe the changes of related vascular and organ pathology, inflammatory factors, and signal pathway proteins, which were verified by statistical analysis.

**Results:**

Low dose IL‐2 can reduce the severity of vascular and bone lesions in collagen‐induced arthritis immune model, and inhibit osteoclast formation in vitro by phosphorylation of nuclear factor‐κB (NF‐κB), which inhibits the receptor activator of NF‐κB ligand effect through c‐Jun N‐terminal kinase (JNK) pathway, and its immunotherapeutic effect depends on the activation of JNK.

**Conclusion:**

It is the first time for us to prove that low dose IL‐2 can inhibit osteoclast formation in collagen‐induced arthritis through the JNK dependent pathway, which will provide the angle and theoretical basis for future immunotherapy of IL‐2.

## Graphical abstract

Low dose interleukin‐2 (IL‐2) can reduce the severity of vascular and bone lesions in the collagen‐induced arthritis immune model. Low dose IL‐2 can inhibit osteoclast formation in vitro by phosphorylation of nuclear factor‐κB (NF‐κB), which inhibits the receptor activator of NF‐κB ligand effect through c‐Jun N‐terminal kinase (JNK) pathway, and its immunotherapeutic effect depends on the activation of JNK.

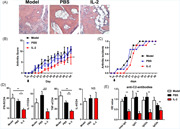

## 1 INTRODUCTION

Rheumatoid arthritis (RA) is one of the most common chronic inflammatory joint disease, which primarily affects the small diarthrodial joints of the body.^1^ At present, the pathological mechanisms of RA have not been fully studied, whereas it is widely believed that in RA autoimmune response leads to the release of proinflammatory and catabolic mediators, which then induces chronic inflammatory in the joint.^2^ The bone resorption induced by osteoclast is hence abnormally enhanced, which leads to the articular damage. Because of the complex pathogenesis, it is still difficult to well treat the RA. Most kinds of drugs for RA, such as methotrexate, are not curative enough because of the heterogeneous curative effects and various kinds of complications.^3^ Thus, novel biologic approaches to cure this disease are sorely needed.

Interleukin‐2 (IL‐2), which belongs to chemokines family, is predominantly produced by T cells and active dendritic cells with multidirectional effects. Study found that low‐dose IL‐2 could activate regulatory T cells (Tregs), which plays a vital role in suppressing the immune response.^4^, ^5^ Thus low‐dose of IL‐2 has been found to have a broad therapeutic potential in many autoimmune and inflammatory diseases, like systemic lupus erythematosus (SLE), sjogren syndrome, and various kinds of tumors.^6^, ^7^, ^8^, ^9^ In recent years it is also found that IL‐2 can suppress the RA. However, the possible mechanisms underlying this function have not been fully understood. Most studies considered that the balance between the Tregs and T helper type 17 cells was the key point regulated by IL‐2 in inhibiting RA.^10^, ^11^, ^12^ In the current study, we found that low dose IL‐2 could also suppress osteoclastogenesis in RA by inhibiting receptor activator of nuclear factor‐κB ligand (RANKL) effect via c‐Jun N‐terminal kinase (JNK) pathway. This novel discovery can help to further understand the mechanism between IL‐2 and RA, and may provide a new strategy for treating the disease as well.

## 2 MATERIALS AND METHODS

### 2.1 Mice

DBA/1J mice (female, 8–10 weeks old) were obtained from Jackson Laboratory. All experiments using mice were performed in accordance with protocols approved by the Institutional Animal Care and Use Committee at The Third Affiliated Hospital of Soochow University.

### 2.2 Induction and treatment of arthritis (collagen‐induced arthritis)

Bovine type II collagen (CII) was extracted and purified from bovine articular cartilage according to established protocols. To obtain the emulsified CII, an equal volume of complete Freund's adjuvant (CFA) with heat‐denatured mycobacterium (4 mg/ml) (Chondrex, LLC) was used. DBA/1 mice were immunized via intradermally injecting 50 μl of emulsion (CII 100 µg/mouse) at the base of the tail. Mice received a single intravenous injection of IL‐2 on Day 14 after immunization to determine the intervention effects.

### 2.3 Evaluation of clinical arthritis

Clinical symptoms of arthritis were evaluated every 2–3 days to determine the incidence of arthritis. Each mouse paw was evaluated and scored by 0–4 scoring system.^13^, ^14^, ^15^, ^16^ The claw scores were added to get the single mouse score, and the maximum score of each animal was 16. The scores of each limb were: 0, no sign; 1, mild swelling limited to tarsal or ankle joint; 2, mild swelling from ankle to tarsal; 3, moderate swelling from ankle to metatarsal joint; 4, severe swelling surrounding ankle, foot, finger, or limb stiffness.

### 2.4 Cytokine analysis

T cells were isolated from spleen and lymph nodes of arthritis mice 60 days after CII injection. They were stimulated in vitro with PMA (50 ng/ml) and ionomycin (500 ng/ml) for 5 h, as well as brefeldin A (10 μg/ml; all from Calbiochem) for 4 h. The expression of IL‐4, IL‐17, interferon‐gamma (IFN‐γ), and tumor necrosis factor‐alpha (TNF‐α) in the cells were detected by flow cytometry.

### 2.5 Osteoclasts generation

For mice experiment, bone marrow or spleen derived CD11b+ cells (OCPs) from DBA2 mice were stimulated for 4 days with rm‐M‐CSF (30 ng/ml) and rm‐RANKL (50 ng/ml) (R&D Systems). Tartrate‐resistant acid phosphatase (TRAP) (Sigma) was used to stain cells according to the manufacturer's instructions, and TRAP+ cells were enumerated under microscopy.

### 2.6 Western blot analysis

Proteins were extracted from harvested cells, and their concentration was determined by the bicinchoninic acid assay (pierce). Protein samples (30 µg) were resolved by sodium dodecyl sulfate‐polyacrylamide gel electrophoresis and transferred to a polyvinylidene difluoride membrane. The following antibodies were used: anti‐mouse RANKL (FL‐317; Santa Cruz), nuclear factor‐κB (NF‐κB) P50 (ab32360; Abcam), NF‐κB P65 (#3033; Cell signaling), JNK (sc‐7345; Santa Cruz), p‐JNK (sc‐6254; Santa Cruz), ERK (sc‐514302; Santa Cruz), p‐ERK (sc‐81492; Santa Cruz). The results were visualized with Kodak autoradiography film (Kodak XAR film).

### 2.7 Statistical analysis

For comparison of treatment groups, we performed unpaired *t* tests (Mann–Whitney), paired *t* tests, and one‐way or two‐way analysis of variance (where appropriate) methods. Percent comparisons were made using the *χ* ^2^ test. All statistical analyses were performed using GraphPad Prism Software (version 4.01). The *p* < .05 is considered as statistically significant.

## 3 RESULTS

### 3.1 Low dose IL‐2 reduces the severity of vascular and bone lesions in collagen‐induced arthritis immune model

**Figure 1:**
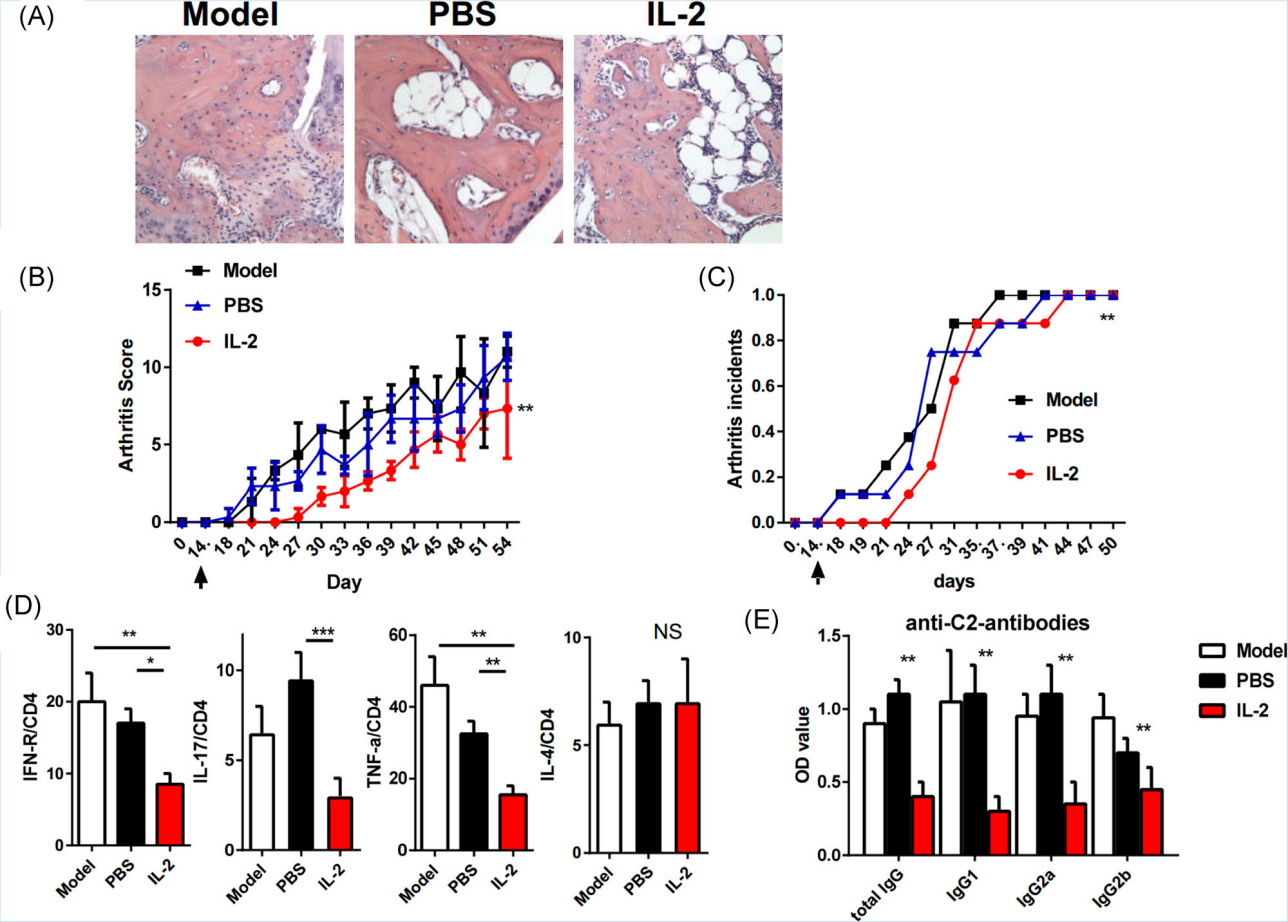
low dose IL‐2 reduces the incidence rate and severity of CIA in vivo. (A) The typical histological changes of the joints in each group on the 56th day after immunization. Joint removal, fixation, hematoxylin and eosin staining. Original magnification ×100; (B) severity of CIA in untreated mice, PBS treated mice, and low‐dose IL‐2 treated mice; (C) incidence of CIA in recipient mice over time, and *p* value was compared with that of untreated CIA mice; (D) cytokines were detected by flow cytometry on the 50th day; (E) anti‐c2 specific IgG subsets were collected from mice serum on the 50th day after immunization and determined by enzyme‐linked immunosorbent assay. *p ≤ .05, **p ≤ .01, ***p ≤ .001. Each group included five mice. All data were from the mean ± *SEM* of three independent experiments. CIA, collagen‐induced arthritis; IgG, immunoglobulin G; IL, interleukin; PBS, phosphate‐buffered saline

We used collagen‐induced arthritis (CIA) model to determine the immunomodulatory effect of low‐dose IL‐2 in autoimmune arthritis. We observed a significant delay in disease onset and ease in arthritis severity scores following injection of IL‐2 (1 × 10^5^ IU/3 days) on Day 14 after CII/CFA immunization. The histological analysis of the whole ankle showed that compared with the control group, the destruction of bone and cartilage in the IL‐2 treatment group were significantly reduced (Figure 1A). The CIA incidence and severity was monitored every 3–5 days after IL‐2 injection. Consistent with Figure 1A, WE observed that IL‐2 treatment markedly decreased the incidence of CIA comparing with Model group (Figure 1B,C). Previous studies have shown that the production of cytokines may be involved in the pathogenesis of arthritis.^12^, ^14^ To determine whether low‐dose IL‐2 can inhibit CIA by controlling the production of cytokines, we studied the mechanism of CIA reduction. We found that the percentage of proinflammatory cytokines such as IFN‐γ, IL‐17, TNF‐α secreted by spleen cells was significantly reduced by injecting IL‐2 into CIA mice, besides, no difference was observed in IL‐4 expression between IL‐2 or PBS treatment (Figure 1D). In addition, injection of IL‐2 into mice significantly inhibited immunoglobulin G 1 (IgG1), IgG2a, and IgG2b (Figure 1E). These results showed that low dose IL‐2 injection reduced the incidence rate and severity of CIA mice.

### 3.2 Low dose IL‐2 can inhibit osteoclast formation in vitro

**Figure 2:**
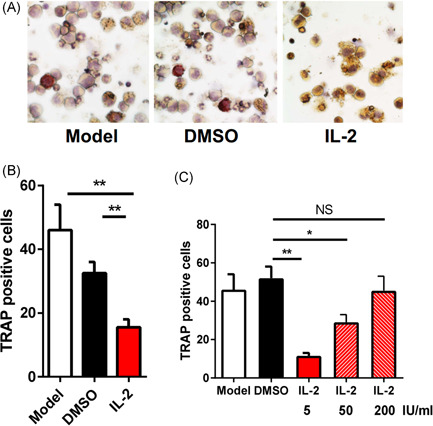
low dose IL‐2 inhibits osteoclast formation in vitro. M‐CSF (30 ng/ml) and RANKL (50 ng/ml) were used to stimulate CD11b+ from bone marrow and cultured with PBS and IL‐2 (5 IU/ml) for 4 days, then TRAP staining was performed. (A and B) representative micrographs of TRAP+ cells in the culture plate; (C) CD11b+ cells were cultured with different doses of IL‐2, and the production of TRAP+ cells was analyzed by cell count. All data were from the mean ± *SEM* of three independent experiments. DMSO, dimethyl sulfoxide; IL, interleukin; PBS, phosphate‐buffered saline; RANKL, receptor activator of the nuclear factor‐κB ligand; TRAP, tartrate‐resistant acid phosphatase

Osteoclast formation is an important factor in the development of RA. To determine whether low‐dose IL‐2 can inhibit osteoclast formation,^17^, ^18^ we directly compared the inhibitory effect of IL‐2 on osteoclast formation. Compared with dimethyl sulfoxide group, the addition of IL‐2 (5 IU/ml) in rm‐M‐CSF and rm‐RANKL stimulated CD11b+ cell cultures reduced the generation of TRAP+ cells (Figure 2A,B). We also evaluated the antiosteoclast effect of different doses of IL‐2. The results showed that low doses of IL‐2 could inhibit the generation of osteoclasts. However, when higher doses of IL‐2 were added to the culture system, the inhibition ability of IL‐2 was weakened (Figure 2C). The results showed that low‐dose IL‐2 could inhibit osteoclast formation in vitro.

### 3.3 The phosphorylation pathway of NF‐κB is involved in the treatment of collagen‐induced arthritis induced by low dose IL‐2

**Figure 3:**
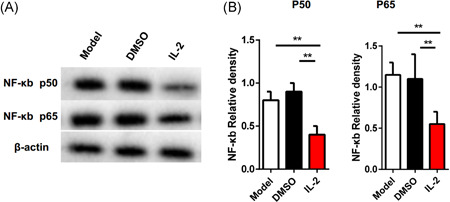
phosphorylation of NF‐κB is involved in the formation of osteoclasts regulated by low‐dose IL‐2. M‐CSF (30 ng/ml) and RANKL (50 ng/ml) were used to stimulate CD11b+ from bone marrow and cultured with PBS and IL‐2 (5 IU/ml) for 4 days, then trap staining was performed. (A) Represented Western blot figure for the levels of NF‐κb p50 and p65 (B) relative level of NF‐κb p50 and p65 activation were determined by Western blot. **p ≤ .01, ***p ≤ .001. All data were from the mean ± *SEM* of three independent experiments. DMSO, dimethyl sulfoxide; IL, interleukin; NF‐κB, nuclear factor‐κB; RANKL, receptor activator of nuclear factor‐κB ligand

It has been well known that RANK/RANKL initiated the NF‐κB pathway, which plays a key role in the early stage of osteoclast formation.^19^ Therefore, we speculate that low dose IL‐2 can inhibit osteoclast formation by regulating these molecular pathways. To test this possibility, we cultured CD11b+ cells with IL‐2 and collected these cells after 4 days. The protein was prepared and purified from the harvested cells, and then analyzed by Western blotting. Compared with the control group, the expression of NF‐κB p65/P50 significantly decreased in the IL‐2 group (Figure 3A,B), indicating that the phosphorylation of NF‐κB participated in the treatment of collagen‐induced arthritis by low dose of IL‐2.

### 3.4 Low dose IL‐2 inhibits RANKL effect through JNK pathway

**Figure 4:**
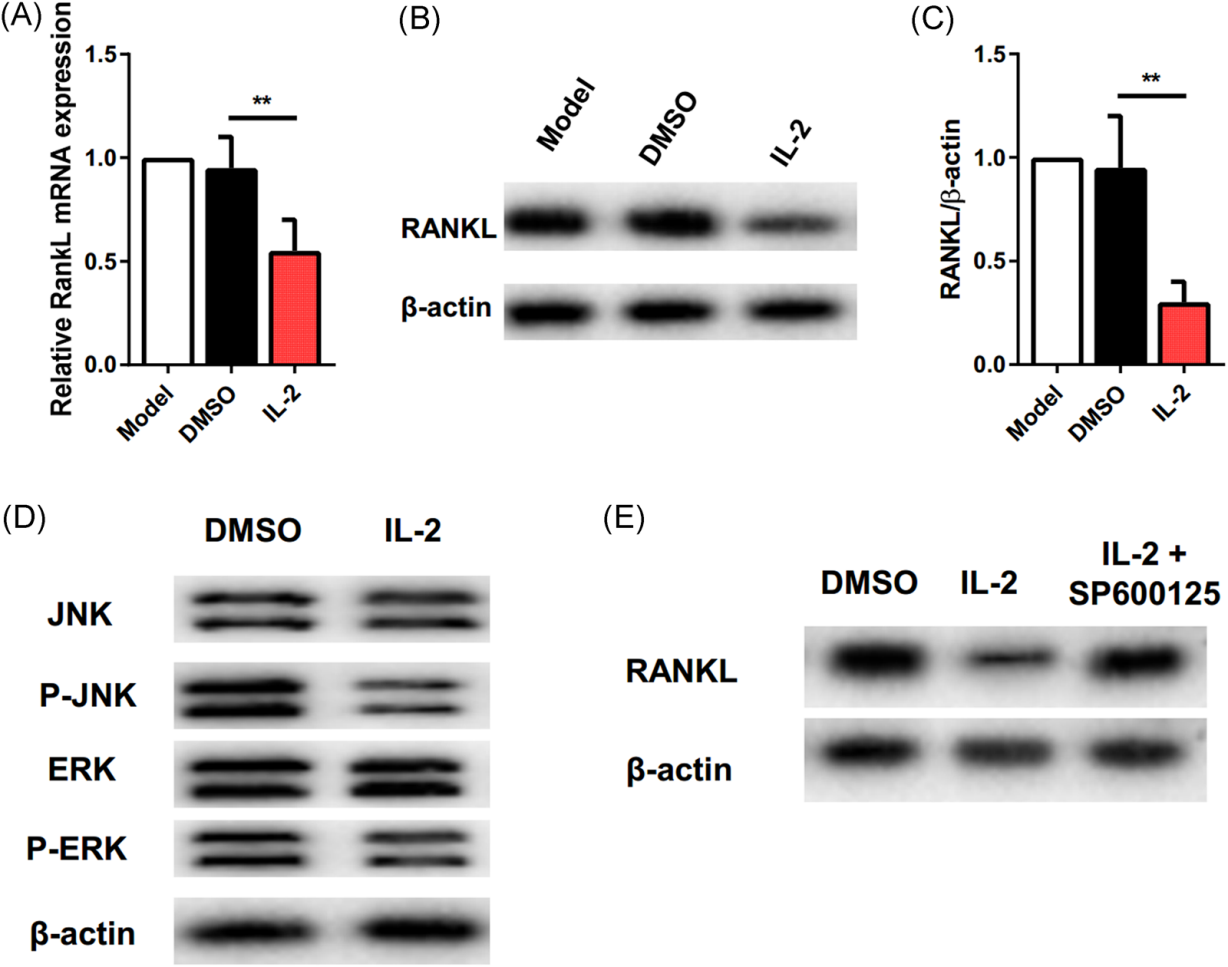
low dose IL‐2 inhibits the RANKL effect in vitro through JNK pathway. (A) Real time PCR showed the relative expression of RANKL gene in different groups in vitro; (B) Western blot showed the expression of RANKL protein in three different groups; (C) quantitative data of relative intensity of RANKL normalized to β‐actin by Western blot; (D) Western blot showed the phosphorylation level of JNK, ERK, and the IL‐2 group between DMSO and actin; (E) in the absence of JNK specific inhibitors, Western blot showed the expression of RANKL. *p ≤ .05, **p ≤ .01. All data were from the mean ± *SEM* of three independent experiments. DMSO, dimethyl sulfoxide;  IL, interleukin; JNK, Jun N‐terminal kinase; RANKL, receptor activator of nuclear factor‐κB ligand

RANKL is an important factor in osteoclast differentiation and activity. To investigate the effect of IL‐2 on RANKL in vitro, we used qRT‐PCR and Western blot to observe the effect of low dose IL‐2 on RANKL in osteoclasts. Compared with the control group, the levels of messenger RNA and protein expressed by RANKL in the IL‐2 group were significantly inhibited (Figure 4A–C). JNK activation is related to osteoclast formation,^20^ Yasuhiko reported that ERK is involved in osteoclast differentiation and cytokine production in the pathogenesis of arthritis.^21^ Therefore, we evaluated the levels of JNK, ERK, and their phosphorylation during osteoclast formation in vitro. IL‐2 could inhibit the phosphorylation of JNK in CD11b+ cells induced by M‐CSF and RANKL for 1 h, but there was no significant change in ERK compared with the control group (Figure 4D). The above data showed that the level of JNK phosphorylation in IL‐2 treatment group decreased significantly. We then analyzed the relationship between JNK and the expression of RANKL. CD11b+ cells were cultured for 4 days by M‐CSF and RANKL in combination with IL‐2. The phosphorylation of JNK was blocked by SP600125 (10 μM), a JNK specific inhibitor. Western blot was used to analyze the expression of RANKL. The results showed that the blocking of JNK activation significantly eliminated the effect of IL‐2 on the RANKL expression (Figure 4E). In conclusion, low‐dose IL‐2 inhibits the RANKL effect through JNK pathway.

### 3.5 The immunotherapeutic effect of low dose IL‐2 depends on the activation of JNK

**Figure 5:**
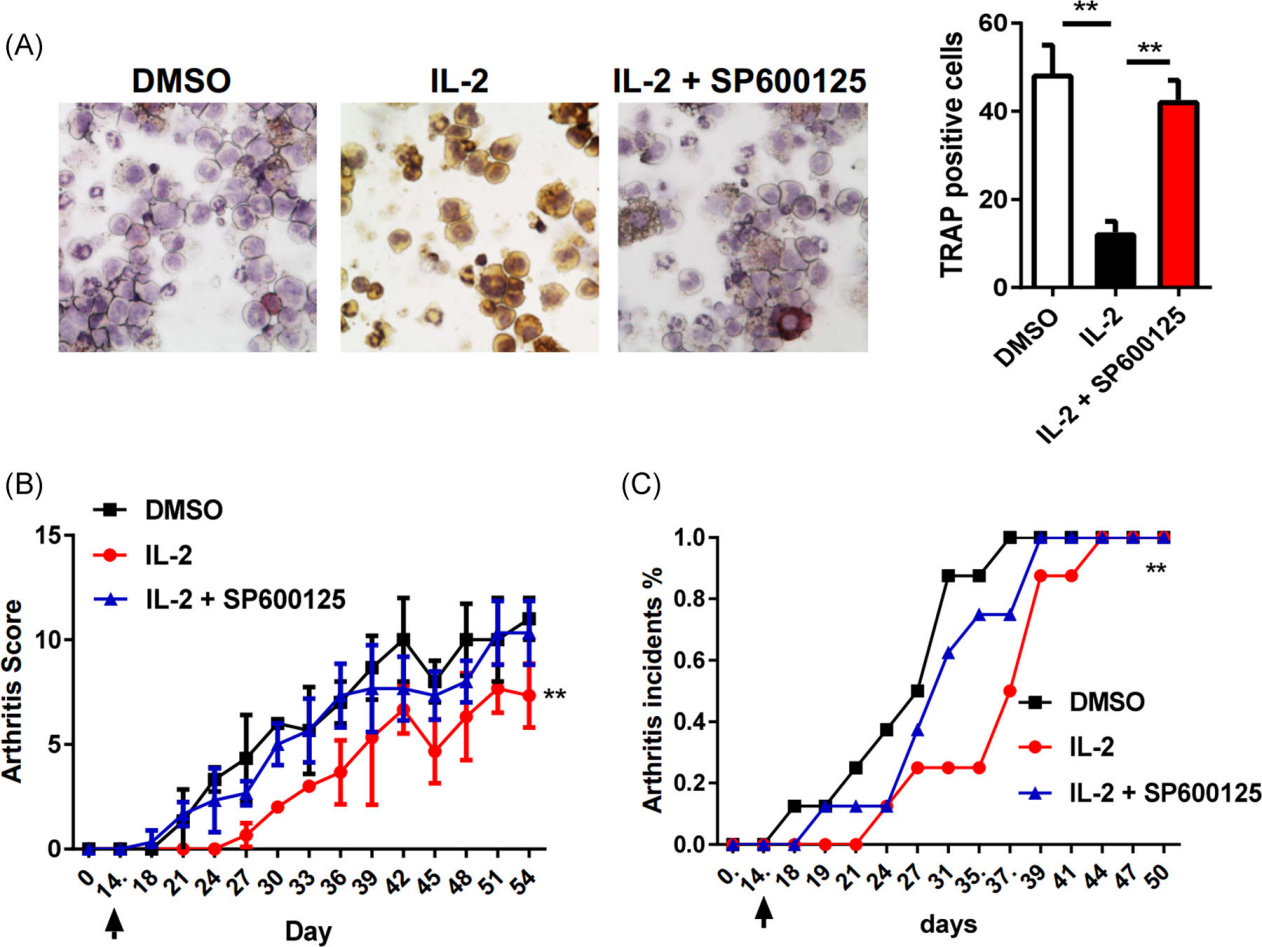
The inhibition of osteoclast production in vitro and in vivo by low‐dose IL‐2 depends on JNK activation. (A) Representative micrographs of TRAP+ cells cultured in different groups. Original magnification ×100; (B) severity of CIA in untreated mice, mice treated with IL‐2 or IL‐2 and SP600125; (C) incidence of CIA in recipient mice treated with IL‐2 or IL‐2 and SP600125 over time. *p* Value was compared with that of untreated CIA mice. *p ≤ .05, **p ≤ .01, ***p ≤ .001. Each group included five mice. All data were from the mean ± *SEM* of three independent experiments. CIA, collagen‐induced arthritis; DMSO, dimethyl sulfoxide; IL, interleukin; JNK, Jun N‐terminal kinase

Next, we examined whether JNK inhibitors could block osteoclast formation in vitro. As shown in Figure 5A, low‐dose IL‐2 inhibited the formation of TRAP+ cells in CD11b+ cells. However, compared with the IL‐2 alone group, JNK inhibitor improved the number of TRAP+ cells. In addition, to determine the activation of JNK in vivo, SP600125 (30 mg/kg iv) was injected on the same day when IL‐2 was injected into the arthritis model. We observed that the injection of IL‐2 significantly blocked the incidence rate of arthritis, and the injection of SP600125 almost completely blocked the role of JNK in vivo (Figure 4B,C). The results showed that the effect of low dose IL‐2 in inhibiting angiogenesis and osteoclast production both in vitro and in vivo highly depended on JNK activation.

## 4 DISCUSSION

IL‐2 is mainly produced by CD4+ T cells and activated dendritic cells and has many functions. High dose of IL‐2 can promote the proliferation of effector T cells, while low dose of IL‐2 can activate Tregs, indicating that IL‐2 has a wide therapeutic potential in many autoimmune and inflammatory diseases.^22^ Clinical studies have confirmed the safety of low‐dose IL‐2 in therapying various autoimmune and inflammatory chronic diseases (including RA, ankylosing spondylitis, SLE, and psoriasis).^4^ In this study, low dose IL‐2 injection can reduce spleen cells in CIA mice secreting proinflammatory cytokines such as IFN‐γ, IL‐17, and TNF‐α, as well as IgG1, IgG2a, and IgG2b. This way reduces the incidence rate of CIA and alleviated the severity of the disease. Therefore, IL‐2 plays an antiinflammatory role, while it will bring less side effects than hormones.

A series of studies have shown the pathogenesis of vascular lesions in RA, including changes in the density of synovial vessels and endothelial cell proliferation. For example, the number of synovial vessels was found to be related to synovial cell proliferation, monocyte infiltration, and joint tenderness index.^23^ Another group noted that although infiltration of monocytes around blood vessels and increased thickness of synovial lining was observed in inflammatory and noninflammatory joint tissues of RA patients, vascular proliferation could only be found in inflammatory joint tissues.^24^ In this study, we found that low‐dose IL‐2 therapy can reduce the vascular lesions in RA, and the underlying mechanism can be explored through further research.

JNK signal plays an important role in the regulation of osteoclast apoptosis, formation, and differentiation related to immune diseases.^25^, ^26^, ^27^ Bone marrow‐derived macrophages isolated from JNK1 knockout mice showed decreased osteoclast differentiation and bone resorption activity.^26^ In addition, the treatment of sp600125, a JNK specific inhibitor, leads to the damage of JNK signal transduction, which makes the antiapoptotic effect of RANKL/RANK/TRAF6 signal transduction in osteoclasts disappear.^25^ It is suggested that the JNK/c‐Jun signaling pathway is involved in the antiapoptosis process of mature osteoclasts induced by RANKL. Even if RANKL continues to exist, blocking JNK activity in the prefusion phase of osteoclasts will lead to the reversal of TRAP+ cells (representing prefusion osteoclasts) to TRAP− cells (representing osteoclast precursors), proving that the JNK pathway is necessary to maintain osteoclast commitment.^28^ We found that low‐dose IL‐2 inhibited osteoclast formation in collagen arthritis through the JNK dependent pathway, which may reflect its direct inhibition on the RANKL pathway of osteoclast and indirect inhibition on other cell types.

The traditional treatment of RA includes hormone combined with an immunosuppressant, but in the previous extensive application, there are some unavoidable problems: (1) most of the treatments can only alleviate symptoms, but cannot fundamentally solve the problem; (2) the treatment process is long, and some patients have difficulties in compliance and economy; 3. the long‐term side effects are large, and the long‐term effects of hormone and immunosuppressant will lead to poor immunity, infection, and other complications. In contrast, low‐dose IL‐2 therapy is designed to restore the function of damaged T cells, with fewer side effects. It can repair the stable immune balance system and bring new hope for patients suffering from the disease for a long time. Under the condition of "Precision medicine" that has been widely advocated in recent years, low‐dose IL‐2 may show great advantages.

There are still some limitations in the current situation of related IL‐2 immunotherapy: (1) the in vivo potency of IL‐2 immunotherapy needs to be further verified due to its instability and short half‐life; (2) there are individual differences in the treatment, which requires strict standardization of treatment frequency and times. Therefore, the further improvement of this therapy needs more in‐depth research. Inconclusion, in this study we have demonstrated for the first time that low‐dose IL‐2 can inhibit osteoclast formation in collagen arthritis through JNK dependent pathway, which will provide a perspective and theoretical basis for the future immunotherapy.

## CONFLICT OF INTERESTS

The authors declare that there are no conflict of interests.

## AUTHOR CONTRIBUTIONS

Yunjie Lu and Xiaoliang Sun conceived and designed the research; Han Sun, Yong Zhao, and Kun Wang performed the experiments and analyzed the data; Han Sun, Yong Zhao, Kun Wang, Li Zhu, Jian Dong, and Jie Zhao prepared the figures and wrote the manuscript; Yimin Wang, Huan Li, Yunjie Lu, and Xiaoliang Sun edited the manuscript; and Yunjie Lu and Xiaoliang Sun revised and approved the final manuscript.

## ETHICS STATEMENT

All experiments using mice were performed in accordance with protocols approved by the Institutional Animal Care and Use Committee at The Third Affiliated Hospital of Soochow University.

## Data Availability

All the data presented here are new and fully accessible.
