# Corrigendum

**DOI:** 10.1002/iid3.685

**Published:** 2022-08-29

In Sun et al.,^1^ there was an error in Figure 1A on page 729.

**Figure 1A:**
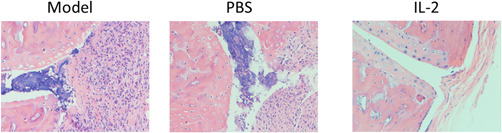


The correct Figure 1A is shown below. The authors state this does not change the scientific conclusions of the article.

The authors apologize for the error.
